# Effects of Acute Long- versus Short-Interval High-Intensity Interval Training on Attention and Psychological States in a Sample of Male and Female Adolescents: A Pilot Study

**DOI:** 10.3390/life13091846

**Published:** 2023-08-31

**Authors:** Maamer Slimani, Mahdi Issaoui, Hela Znazen, Amri Hammami, Nicola Luigi Bragazzi

**Affiliations:** 1 School of Public Health, Department of Health Sciences (DISSAL), Genoa University, 16126 Genoa, Italy; 2 Higher Institute of Sport and Physical Education of Gafsa, University of Gafsa, Gafsa 2112, Tunisia; mahdi.issaoui.tn@gmail.com; 3 Department of Physical Education and Sport, College of Education, Taif University, P.O. Box 11099, Taif 21944, Saudi Arabia; znazen.hela@laposte.net; 4 Higher Institute of Sport and Physical Education of Ksar Said, University of Manouba, Manouba 2010, Tunisia; hammamiamri@hotmail.com; 5 Laboratory for Industrial and Applied Mathematics (LIAM), Department of Mathematics and Statistics, York University, Toronto, ON M3J 1P3, Canada; robertobragazzi@gmail.com

**Keywords:** dose–response, HIIT, cognition, attention, mood

## Abstract

The aim of this study was to assess the effect of acute short- versus long-interval high-intensity interval training (HIIT) on cognitive performance and psychological states in secondary school students. Fifteen secondary school students (nine males and six females: mean age = 16.2 ± 0.4 years, mean Body Mass Index = 21.2 ± 1.5 kg/m^2^, and maximum oxygen uptake = 42.2 ± 5.9 mL/kg/min) participated in the current study. They performed one of the following three sessions in a randomized order: (i) a long-interval HIIT (LIHIIT), (ii) a short-interval HIIT (SIHIIT), and (iii) a control condition (CC). Cognitive performance and perceived exertion were assessed pre and immediately post each condition using the d2 test and the Rating of Perceived Exertion (RPE) tool, respectively. Mood state was quantified using the Brunel Mood Scale (BRUMS) questionnaire immediately post each condition. The findings reported higher concentration performance in the SIHIIT compared to the LIHIIT condition (*p* = 0.043) and the CC (*p* < 0.001) and in the LIHIIT compared to the CC (*p* = 0.023). Moreover, the total count of errors was higher in the CC than in the LIHIIT (*p* = 0.01) and in the SIHIIT conditions (*p* < 0.001) and in the LIHIIT than in the SIHIIT condition (*p* = 0.03). RPE value was higher in the LIHIIT and SIHIIT conditions than in the CC (both *p* < 0.001), whereas no statistically significant difference between LIHIIT and SIHIIT conditions (*p* = 0.24) was found. Regarding the BRUMS, a significant difference between conditions in the fatigue subscale was found, being higher in LIHIIT with respect to SIHIIT (*p* = 0.03) and CC (*p* < 0.05). Vigor differed between conditions, with a higher value than in the LIHIIT (*p* = 0.04) and CC (*p* < 0.001). All the remaining subscales did not significantly differ between conditions (*p* > 0.05). Practitioners may implement short-interval HIIT prior to any tasks that require high levels of visual attention.

## 1. Introduction

Current developments in the arena of exercise–cognition research show that acute physical exercise may be beneficial for people’s cognitive performance and, specifically, attention [1]. The most notable benefits can be observed in the elderly, with physical activity contributing to slowing the rate of cognitive decline associated with aging [2]. Enhanced cognitive abilities, such as improved attention and optimal cognitive performance, can effectively support the processes of psychosocial adaptation by aiding in the adjustment to one’s surroundings and playing a role in achieving higher levels of accomplishment in various academic and social endeavors [3]. The effect of physical activity on cognitive performance varies across age groups and types of exercise, the type of cognitive performance assessed, the intensity and duration of exercise, and the participant’s fitness [1,4]. Although positive results are frequently and clearly documented for children and adult samples, studies with adolescents and young adults are less often conducted and the reported effects are less consistent. For example, Budde et al. [5] and Hogan et al. [6] observed better performance-related outcomes, including attention levels, after an acute bout of exercise, while Hogan et al. [7] and Stroth et al. [8] reported null effects. Altermann and Gropel [9] demonstrated that attentional test performance increased from before to after different interventions including endurance, strength, and coordination exercises.

Moreover, most of the studies assessing the perceptual and cognitive responses to acute exercise bouts have focused on traditional programs of physical activity or simple tasks such as walking or running and coordination exercises [10,11]. Currently, less is known about the acute effects of high-intensity interval training (HIIT) on attention and psychological state given its growing popularity in recent years as an effective, attractive, and time-efficient training mode to improve the health and wellbeing of the general population. HIIT, which alternates bouts of high-intensity exercise with low-intensity recovery periods, is one of the most efficient ways to enhance cardio-metabolic and respiratory functioning [12]. Weston et al. [13] reported in a meta-analysis that HIIT, typically performed at an 85–95% maximal heart rate (%HR_max_), can improve cardiorespiratory fitness by almost double compared to continuous, moderate-intensity training in patients with lifestyle-induced chronic disorders and comorbidities. Additionally, research has highlighted that HIIT elicited positive long-term effects on cognitive performance (i.e., concentration, selective attention, or working memory) and psychological outcomes (i.e., depressed mood, emotional well-being, and sleep quality) in youth [14].

There is an accumulating body of scholarly research on the acute effects of HIIT on cognition and psychological state, even though both the direction and the magnitude of these impacts still have to be elucidated. For instance, it has been reported in a recent meta-analysis that the acute effects of HIIT on inhibitory control are inconsistent with both positive and null effects being found [15]. Some scholars documented positive effects after HIIT sessions compared with single bouts of continuous aerobic [4,11,15] and resistance exercise [4]. In recent systematic reviews and meta-analyses, it has been demonstrated that, on the one hand, acute HIIT interventions with a total time duration between 11 and 20 min or between 21 and 30 min tended to have positive effects on executive function [16,17,18], but, on the other hand, those with a total time of less than 10 min or more than 30 min did not consistently show positive effects. 

To date, the dose–response relationship between HIIT duration and cognition is unknown. However, some recent studies explored the dose–response relation between resistance/aerobic exercise intensity and cognitive performance after exercise. For example, Chang et al. [19] found that acute aerobic exercise for 30 min (i.e., 5 min warm-up, 20 min main exercise, and 5 min cooldown) has beneficial effects on EF, whereas acute exercise for 10 min or 45 min showed negligible effects. Moreover, according to Chang et al. [20], practicing resistance exercise at 100% of 10-repetition maximum (RM) could support lower-level cognitive performance relative to 40% and 70% of 10RM, suggesting a linear trend, whereas 70% of 10RM resulted in better performance-related outcomes on interference and working memory. Therefore, it is paramount for future research to account for the potential impacts of dose–response relations between bouts of acute exercise (in terms of intensity) and cognitive-performance-related outcomes.

It should be emphasized that an acute exercise prescription takes into account various parameters related to exercise itself, from its modality to intensity and duration [20], and even though dose–response relations between exercise intensity (i.e., aerobic and resistance) and cognitive performance have been relatively well described, the precise effects of HIIT duration still have to be thoroughly assessed, especially in adolescent populations. As such, the aim of the present study was to test the effect of acute short- (SIHIIT) versus long-interval HIIT (LIHIIT) on cognitive performance and psychological state in a study involving secondary school students. More specifically, we hypothesized that both short- and long-interval HIIT sessions will improve attention and psychological states with respect to the control condition in our population. We also hypothesized higher concentration performance and a lower total number of errors after the SIHIIT session, as well as lower perceived exertion and higher vigor. 

## 2. Materials and Methods

### 2.1. Participants 

The a priori sample size and power analyses were carried out by means of the freely available G*power software (version 3.1.9.7 for Windows, Universität Düsseldorf: Psychologie, Düsseldorf, Germany), which was utilized in order to determine the appropriate sample size of the trial. Under the scenario of a large effect size of the intervention (with an expected f of 0.40), with the alpha and 1 – beta values being set at 0.05 and 0.80, respectively, with three conditions (SIHIIT, LIHIIT, and a control condition, CC) and two measurements (before and after), and with an expected correlation of 0.70, at least 15 participants should be recruited in the study. 

Fifteen secondary school students (nine males and six females, a mean age of 16.2 ± 0.4 years, a mean Body Mass Index of 21.2 ± 1.5 kg/m^2^, and a maximum oxygen uptake (VO_2max)_ of 42.2 ± 5.9 mL/kg/min) participated in the current study. They performed one of the following three sessions in a randomized order: (i) a LIHIIT, (ii) a SIHIIT, and (iii) a CC. We included participants if they met the following condition(s): (a) not performing high-intensity activities prior to the trial; (b) practicing structured physical activity two times a week; and (c) refraining from the intake of any supplement (e.g., coffee and vitamins) that could affect the results of the study. We excluded any participants who did not meet these criteria. 

The investigation was conducted in accordance with the guidelines of the Declaration of Helsinki and received full clearance from the UNESCO Chair “Health Anthropology Biosphere and Healing Systems” (University of Genoa, Genoa, Italy). All participants and their parental guardians were informed about the objective of the study and gave their informed, written consent to be included in this investigation.

### 2.2. Procedure

The investigation was carried out using a randomized crossover design. It was conducted in four sessions separated by a washout period of 72 h, given that the recommended time for muscle recovery is (at least) 48–72 h. During the first session, participants completed the 20 m multistage fitness test to determine their individual exercise workload or maximal aerobic speed (MAS) and familiarized themselves with the cognitive tests and the psychological questionnaires.

During the second and third sessions (LIHIIT and SIHIIT sessions), all participants responded to the Rating of Perceived Exertion (RPE) questionnaire and completed a cognitive performance test (d2 test) 5 min before and immediately after each condition, in addition to responding to the Brunel Mood Scale (BRUMS) questionnaire.

Both sessions were performed on an outside 300 m track and started with a 10 min warm-up, which consisted of 5 min of moderate-intensity (50% of MAS) running followed by 5 min of dynamic stretching exercises and 3 repetitions of 30 m of accelerations. Then, at the end of each session, participants performed a 10 min cooldown by jogging at low intensity and static stretching.

During the SIHIIT session, participants performed 2 sets of 5 repetitions of individualized distance running lasting 30 s at 110% of MAS, interrupted with 30 s of passive recovery between repetitions and 2 min of active recovery at 40% of MAS between sets. In other words, participants had to run a given controlled distance for 30 s. The distance was marked by two cones (namely, cone 1 and cone 2) in relation to the speed requested. An acoustic signal was provided at the start (cone 1) and at the end of the 30 s period (cone 2). During the LIHIIT session, participants performed three runs lasting 120 s at 90% of their own MAS interrupted with 2 min active recovery, as in the SIHIIT, at 40% of MAS. Of note, each training session lasted approximately 32 min with a work-to-recovery ratio (WRR) of 1, as it is better than other ratios [15,21]. 

During the control session, participants were asked to read a book for approximately 32 min and completed cognitive and psychological tests as during the other conditions.

### 2.3. The 20 m Multistage Fitness Test

The MSFT was conducted by following the instructions provided by Léger et al.’s [22] study. In the extant scholarly literature, it is well known that VO_2max_ can be predicted in adult populations with an intra-class correlation coefficient of 0.90 [23]. When conducting the test, the participants have to run backwards and forwards between two lines 20 m apart, in accordance with the recorded “beep” sound. Runs are considered successful only if shuttles are properly completed. The test starts with an initial velocity of 8 km/h, which is gradually incremented by 0.5 km/h every minute and is halted if the subject does not reach the line (within 2 m) for two consecutive ends, after being properly advised. MAS is calculated as the speed of the last successfully completed stage, associated with VO_2max_ for the shuttle run test. As previously mentioned, VO_2max_ is computed by utilizing the method developed by Léger et al. [22].

### 2.4. Attention Assessment

The cognitive performance d2 test was delivered in its paper-and-pencil format. It was utilized to assess the participants’ levels of concentrated visual attention from a quantitative standpoint [24], that is to say, it was leveraged to capture the complex series of cognitive processes that drive the selection of important information from the environment. It consists of a grid of 14 rows, each one including 47 characters (the letter d or p, with one to four dashes above and below each letter). Participants had to scan each line and mark only the letter d with two dashes. 

The primary outcome measures derived from the d2 test include concentration performance and the overall count of errors. Concentration performance is calculated by tallying the correctly crossed-out d2 symbols and subtracting the incorrectly crossed-out symbols. Meanwhile, the total number of errors is determined by summing up instances where participants fail to identify a d2 symbol and cases of mistakenly crossing out non-d2 symbols. It is important to highlight that, if there is an increase or decrease in concentration performance after the intervention as compared to the baseline, it indicates whether the level of visual attention is deemed satisfactory or not up to par, respectively.

### 2.5. Rating of Perceived Exertion (RPE)

At the commencement and completion of every session, each participant underwent a thorough assessment using the RPE scale to gauge their personal perception of exertion. This scale encompassed a spectrum from 0, signifying “no perceived exertion” (akin to rest), to 10, representing “maximal perceived exertion” (indicative of the most strenuous exercise ever undertaken) [25].

### 2.6. Mood

To assess participants’ mood state, the BRUMS was used [26] by asking “How do you feel right now?”, immediately at the end of each session. The BRUMS consists of 6 subscales (fatigue, anger, vigor, confusion, depression, and tension), each consisting of 4 relevant items, totaling 24 items, and is graded on a 5-point Likert scale (0 = not at all, 4 = extremely). As such, the raw score of each subscale ranges from 0 to 16.

### 2.7. Statistical Analysis

Summary statistics were generated by calculating the means and standard deviations for each of the parameters under investigation. Due to the limited sample size employed, the normality of data distribution underwent examination through the Shapiro–Wilk test. Based on the test results, comparisons were made using one- and two-way analysis of variance (ANOVA) or their non-parametric counterparts. These analyses aimed to identify any differences in (a) pre- and post-intervention measurements and (b) among different conditions. For evaluating the main and interaction effects, the effect size (ES) was computed using partial eta-squared [27], wherein ES values below 0.06 were deemed small and those exceeding 0.14 were considered large. 

All statistical analyses were conducted using the commercial software “Statistical Package for Social Sciences” (SPSS for Windows, version 24.0, IBM, Armonk, NY, USA). Findings reaching a significance threshold of 0.05 were considered statistically significant.

## 3. Results

For the concentration performance, there was a statistically significant main effect of time (F_(1,42)_ = 221.28; *p* < 0.001; ES = 0.84), with higher concentration performance values post-exercise compared with pre-exercise. A main effect of the intervention (F_(1,42)_ = 13.79; *p* < 0.001; ES = 0.39) was found. An interaction (time × intervention) effect was also observed (F_(1,42)_ = 66.79; *p* < 0.001; ES = 0.76). Post hoc analyses of pairwise comparisons revealed that the post-exercise value was higher in the SIHIIT compared to the LIHIIT condition (*p* = 0.043) and the CC (*p* < 0.001) and in the LIHIIT compared to the CC (*p* = 0.023) (Table 1).

Concerning the total count of errors, a main effect of time (F_(1,42)_ = 274.12; *p* < 0.001; ES = 0.86) and a main effect of the intervention (F_(1,42)_ = 16.27; *p* < 0.001; ES = 0.43) were found (Table 1). An interaction (time × intervention) effect (F_(1,42)_ = 89.74; *p* < 0.001; ES = 0.81) was also observed. Moreover, post hoc analyses of pairwise comparisons revealed that the total number of errors value was higher in the CC than in the LIHIIT (*p* = 0.01) and in the SIHIIT conditions (*p* < 0.001) and in the LIHIIT than in the SIHIIT condition (*p* = 0.03).

The RPE values differed between interventions (F_(1,42)_ = 125.68; *p* < 0.001; ES = 0.85). A main effect of time (F_(1,42)_ = 796.83; *p* < 0.001; ES = 0.95) was observed, with higher values being recorded post-exercise compared with pre-exercise (*p* < 0.05). Also, an interaction (time × intervention) effect (F_(1,42)_ = 140.23; *p* < 0.001; ES = 0.87) was found. A post hoc analysis of pairwise comparisons revealed that the RPE value was higher in the LIHIIT and SIHIIT conditions than in the CC (both *p* < 0.001), whereas no statistically significant difference between LIHIIT and SIHIIT conditions (*p* = 0.24) was found (Table 1).

Regarding the BRUMS, the one-way ANOVA showed a significant difference between conditions in the fatigue subscale (F_(1,42)_ = 48.67; *p* < 0.001; ES = 0.67), being higher in LIHIIT with respect to SIHIIT (*p* = 0.03) and CC (*p* < 0.05). Vigor differed between conditions (F_(1,42)_ = 14.68; *p* < 0.001; ES = 0.41), with a higher value than in the LIHIIT (*p* = 0.04) and CC (*p* < 0.001). All the remaining subscales did not significantly differ between conditions (*p* > 0.05) (Table 2).

## 4. Discussion

To the best of the authors’ knowledge, this is the first investigation that aimed to compare the effect of acute long- versus short-interval HIIT on cognition (attention) and psychological states (mood). As expected, the results of this study indicated that both long- and short-interval HIIT have a positive effect on attention. However, a greater increase in concentration performance and a decrease in the total number of errors were reported after the SIHIIT than the LIHIIT. This beneficial effect may be explained by the positive improvement of mood subscales, specifically the increase in vigor and the decrease in fatigue following SIHIIT when compared to LIHIIT.

A number of investigations studied the effect of acute HIIT on cognitive performance using different conditions and settings in terms of exercise volume, time:rest ratio, and cognitive tests. For instance, a systematic review of the literature on the effect of acute HIIT on EF published in March 2021 screened 521 studies, retaining 24 studies from twelve countries [16]. The authors reported that 61% of the outcomes from the included studies suggested that EF was positively affected by acute HIIT. Similarly, Kujach et al. [28] reported that 8 min of HIIT with a time:rest ratio of 1 (30 s:30 s) had a positive effect on executive function, as assessed by the Stroop test, in a sample of sedentary participants. In contrast, other studies failed to identify the beneficial effects of acute HIIT on cognitive performance [29,30]. The contradiction between the findings of the studies may depend on many factors, such as the HIIT volume, protocol of HIIT implemented, and rest interval mode. 

The positive improvement of cognitive performance following HIIT is possibly due to the alterations of physiological parameters (such as heart rate and lactate) and neurochemicals in the brain (such as cortisol; catecholamines (noradrenaline and dopamine); brain-derived neurotrophic factor (BDNF), a biomarker associated with cognitive performance; and blood flow alterations), which in turn may increase the attentional resources of the individual engaged in cognitive performances [31,32,33,34,35,36,37]. In this regard, some authors reported that serum BDNF was higher following HIIT than after continuous high-intensity exercises [35]. In more detail, the neuronal enhancement in terms of BDNF may be due to the higher increase in brain H_2_O_2_ and TNF-α levels, which may both activate the signaling of the peroxisome proliferator-activated receptor (PPAR) after HIIT [35,36]. For instance, short intervals and moderate periods of intense exercise, as were included in the current study, have been shown to directly improve cognitive performance in conjunction with elevated BDNF levels and peripheral catecholamines [38]. In addition, other studies have also suggested that acute HIIT increased the activation and oxygenation of the prefrontal cortex and other brain regions associated with cognitive performance [39,40,41]. When comparing long- versus short-interval HIIT and different WRRs of HIIT on cognitive performance, it has been shown that the WRR and work interval of HIIT may moderate the relation between acute HIIT and cognitive inhibition [41] and attention. Shorter intervals and smaller WRRs (e.g., 30 s:30 s) may have a greater effect than longer intervals (120 s:120 s) and larger WRRs (e.g., 60 s:30 s) [41]. On the one hand, to examine the effects of short-interval (≤30 s) HIIT on execution function, a previous study reported that ten sets of 10 s of sprint exercise at maximal running pace interspersed with 50 s of active recovery/walking [42] and an actual exercise time of 10 min increase response time on the Stroop test by 5.4% [42]. Slusher et al. [36] reported that 10 sets of pedaling at the maximal intensity on a cycle ergometer for 20 s against resistance of 5.5% of the participant’s body weight and then undergoing 10 s of active recovery (5 min of actual exercise) significantly increased the number of categories completed (ES = 0.32) and correct responses (ES = 0.23) and decreased the total errors (ES = 0.23) and non-perseverative errors (ES = 0.30), as assessed by the Wisconsin card sorting task, when compared with the control condition. Similar results were also reported in the study of Kujach et al. [28], who showed that eight sets of 30 s of cycling bouts followed by 30 s of passive recovery for a total time of 8 min had a significant effect of reaction time compared with the CC. Another study examined the effect of 6 min of HIIT with a protocol consisting of 10 sets of 6 s of cycling bouts against resistance of 7.5% of the participant’s body weight followed by 30 s of passive recovery on the Go/No-Go task performance [43]. The authors showed that 6 min of HIIT under normoxia and moderate hypoxia did not impair reaction time and/or inhibitory-based cognitive performance. Accordingly, similar findings were also reported in the study of Wilke et al. [44] regarding the effect of 15 min of functional whole-body exercises carried out in a circuit format with 20 s of active recovery and 10 s of recovery.

Regarding the impact of long-interval (≥2 min) HIIT on cognitive performance, Quintero et al. [45] reported that four sets of 4 min of running at 85–95% HR_max_ followed by 4 min of active recovery at 75–85% HR_max_ increased CP (ES = 0.44) with no significant difference compared with the control condition. This latest finding was contradictory when compared to our results: this discrepancy may be explained by accounting for the difference in session volume. Taken together with our study findings, it seems that acute short-interval (≤30 s) HIIT with a WRR of 1 for 8/12 min may be appropriate as a time- and interval-efficient exercise regime for the improvement of attention and executive function in youth. This could be explained by taking into account that this type of exercise leads to increased cortical activation in the prefrontal cortex [28], which in turn facilitates executive function and improves attention.From an exercise psychology perspective, it has been reported that a single session of HIIT with WRRs of 1 min:1 min and 10 s:10 s may lead to a significant decrease in the Profile of Mood States, which is represented by increases in fatigue and tension and confusion levels and a decrease in vigor [46,47]. In contrast, Martinez et al. [48] showed a greater positive affective response following short-interval HIIT (30 s) and moderate-interval HIIT (60 s) when compared with long-interval HIIT (120 s). Thus, HIIT based on longer exercise periods and imbalanced ratios (e.g., 1:0.5) may result in lower affective responses [48,49].

Concerning the strengths and limitations of the present study, the current investigation should be considered as an initial pilot study with preliminary findings. Further larger studies should replicate and corroborate our findings, exploring sex- and gender-specific differences as well, for which our study was underpowered. Moreover, different HIIT protocols, in terms of intervals and WRRs, should be more comprehensively appraised. An important aspect to highlight is that we successfully identified distinct alterations in mood and cognitive performance. However, the duration of these changes and their consistency over time remain uncertain. Future investigations should delve into the lasting nature of these changes and their possible significance in relation to extended advantages.

## 5. Conclusions

In conclusion, the present study added new knowledge for coaches and physical education teachers about the acute effect of HIIT on attention and psychological states. The findings reported that single sessions of SIHIIT can have greater effects on concentration performance than LIHIIT in secondary school students. Practitioners may implement short-interval HIIT prior to any tasks that require high levels of visual attention. However, based on the previously mentioned shortcomings, further high-quality research in the field is warranted. 

## Figures and Tables

**Table 1:** Values of concentration performance, total number of errors, and RPE broken down according to the condition.

Variable		LIHIIT (Mean ± SD)	SIHIIT (Mean ± SD)	CC (Mean ± SD)	Statistical Significance (Time × Intervention)
Concentration performance	Before	218.13 ± 15.31	215.13 ± 15.49	214.59 ± 16.93	*p* < 0.001
	After	244.53 ± 16.03	274.73 ± 17.01	218.00 ± 17.15	
Total number of errors	Before	79.86 ± 13.74	82.86 ± 14.36	83.26 ± 16.87	*p* < 0.001
	After	53.46 ± 14.41	23.26 ± 14.59	81.46 ± 17.28	
RPE	Before	0.86 ± 0.51	0.80 ± 0.56	0.73 ± 0.59	*p* < 0.001
	After	8.66 ± 1.44	8.13 ± 1.30	1.60 ± 0.73	

CC: control condition, LIHIIT: long-interval high-intensity interval training, RPE: Rating of Perceived Exertion, SIHIIT: short-interval high-intensity interval training.

**Table 2:** Scales of the BRUMS broken down according to the condition.

Variable	LIHIIT	SIHIIT	CC	Statistical Significance
Anger subscale	6.06 ± 1.75	5.73 ± 1.86	5.46 ± 1.92	*p* = 0.6
Confusion subscale	8.13 ± 2.85	7.20 ± 2.17	6.13 ± 1.59	*p* = 0.4
Depression subscale	5.20 ± 1.93	4.93 ± 1.38	4.60 ± 1.35	*p* = 0.6
Fatigue subscale	13.06 ± 2.40	11.46 ± 2.02	6.20 ± 1.42	*p* < 0.001
Tension subscale	6.60 ± 2.44	6.26 ± 2.25	5.66 ± 2.02	*p* = 0.7
Vigor subscale	10.73 ± 2.01	12.26 ± 2.34	8.26 ± 1.70	*p* < 0.001

CC: control condition, LIHIIT: long-interval high-intensity interval training, SIHIIT: short-interval high-intensity interval training.

## Data Availability

Data generated by the investigation are within the manuscript. Further data can be obtained upon request to the Corresponding Author.
