# Correction: Slimani et al. Effects of Acute Long-Versus Short-Interval High-Intensity Interval Training on Attention and Psychological States in a Sample of Male and Female Adolescents: A Pilot Study. *Life* 2023, *13*, 1846

**DOI:** 10.3390/life14121635

**Published:** 2024-12-10

**Authors:** Maamer Slimani, Mahdi Issaoui, Hela Znazen, Amri Hammami, Nicola Luigi Bragazzi

**Affiliations:** 1 School of Public Health, Department of Health Sciences (DISSAL), Genoa University, 16126 Genoa, Italy; 2 Higher Institute of Sport and Physical Education of Gafsa, University of Gafsa, Gafsa 2112, Tunisia; mahdi.issaoui.tn@gmail.com; 3 Department of Physical Education and Sport, College of Education, Taif University, P.O. Box 11099, Taif 21944, Saudi Arabia; znazen.hela@laposte.net; 4 Higher Institute of Sport and Physical Education of Ksar Said, University of Manouba, Manouba 2010, Tunisia; hammamiamri@hotmail.com; 5 Laboratory for Industrial and Applied Mathematics (LIAM), Department of Mathematics and Statistics, York University, Toronto, ON M3J 1P3, Canada; robertobragazzi@gmail.com

**Addition of an Author**

“Mahdi Issaoui” was not included as an author in the original publication [1]. In addition to affiliation 2, the updated affiliation and front note should include:

2 Higher Institute of Sport and Physical Education of Gafsa, University of Gafsa, Gafsa 2112, Tunisia.

^†^  These authors contributed equally to this work (co-first authors).

The corrected Author Contributions statement appears here.

**Author Contributions:** Conceptualization, M.S. and N.L.B.; methodology, M.S. and N.L.B.; software, M.S.; validation, M.S.; formal analysis, M.S. and N.L.B.; investigation, M.S.; resources, M.S.; data curation, M.S.; writing—original draft preparation, M.S., H.Z., A.H. and N.L.B.; writing—review and editing, M.S., M.I., H.Z., A.H. and N.L.B.; project administration, M.S.; funding acquisition, M.S. and N.L.B. All authors have read and agreed to the published version of the manuscript.

The authors state that the scientific conclusions are unaffected. This correction was approved by the Academic Editor. The original publication has also been updated.
